# IPSS-M downstaging before transplantation does not improve the prognosis of patients with myelodysplastic neoplasms

**DOI:** 10.1038/s41409-026-02845-w

**Published:** 2026-03-31

**Authors:** T. Richardson, D. Schütte, P. Gödel, C. von dem Bongart, C. Burkhard-Meier, E. Lorsy, K. Kreuzer, L. Frenzel, M. Hallek, U. Holtick, C. Scheid

**Affiliations:** Department I of Internal Medicine, University of Cologne, Faculty of Medicine and University Hospital Cologne, Cologne, Germany

**Keywords:** Myelodysplastic syndrome, Stem-cell therapies

## Abstract

Allogeneic hematopoietic cell transplantation (ASCT) is the only curative option for patients with myelodysplastic syndromes (MDS), but whether cytoreductive pretreatment and molecular “downstaging” according to the IPSS-M improves outcomes remains unclear. We retrospectively analyzed 128 consecutive adults with MDS who underwent ASCT grouped as frontline transplantation (*n* = 87) or pretreated before transplant (*n* = 41). Median bone marrow blasts at diagnosis were 12% vs. 10%. IPSS-M was calculated at diagnosis and immediately before transplant using cytogenetic and next-generation sequencing data. IPSS-M improved in 26% of frontline and 34% of pretreated patients, was unchanged in 41% and 34%, and worsened in 30% and 32%, respectively. After a median follow-up of 17.3 months, overall survival (OS), relapse-free survival (RFS) and graft-versus-host disease relapse-free survival (GRFS) were superior with frontline transplantation (median OS 112.6 vs 14.0 months, *p* = 0.03, median RFS 61.0 vs 8.9 months, *p* = 0.007 and median GRFS 13.3 vs 5.3 months, *p* = 0.004). However, in a landmark analysis starting at the time of transplantation, the difference in OS was no longer statistically significant. Non-relapse mortality was significantly higher after pretreatment (*p* = 0.018). Pretransplant cytoreduction did not improve post-transplant outcomes despite modest IPSS-M improvements, supporting molecular-risk–guided timing and early donor identification rather than treatment aimed at IPSS-M downstaging.

## Introduction

Myelodysplastic neoplasms (MDS) are clonal disorders of hematopoietic stem and progenitor cells with heterogeneous genetic backgrounds and variable risk of transformation into acute myeloid leukemia (AML) [1]. Allogeneic stem cell transplantation (ASCT) remains the only curative option for patients with or high-risk disease [2]. The current WHO 5th edition subdivides MDS with increased blasts into IB-1 (5–9%) and IB-2 (10–19%), while the International Consensus Classification (ICC) introduces MDS/AML for 10–19% blasts without AML-defining genetics, reflecting the biological continuum between MDS and AML [1, 3].

The role of pretransplant cytoreduction before ASCT remains controversial [4]. Elevated blast counts at transplant are associated with inferior survival [5], yet attempts to downstage disease with chemotherapy or hypomethylating agents (HMAs) can increase toxicity and promote resistant clones [6–8]. Retrospective studies have produced inconsistent results [9–11]. Leveraging the Revised International Prognostic Scoring System (IPSS-R) [12], Scheid et al. recently reported in an EBMT registry analysis that improvement in IPSS-R before transplantation conferred a modest survival advantage only in patients receiving intensive chemotherapy, whereas worsening scores predicted much poorer outcomes irrespective of the therapy chosen [13]. Meanwhile, prospective data from the ACROBAT trial (NCT04184505) that randomizes frontline transplantation or HMA followed by transplantation in higher-risk MDS are pending.

Parallel advances in genomic profiling have made molecular diagnostics a routine part of MDS evaluation and most patients harbor at least one, in median three, characteristic somatic mutations [14, 15]. Building on this knowledge, the Molecular International Prognostic Scoring System (IPSS-M) incorporates 31 mutations into a single model that surpasses IPSS-R in predicting overall survival (OS), relapse-free survival (RFS), and AML progression [16]. Subsequent studies validated its use in transplanted cohorts: Gurnari et al. demonstrated that IPSS-M outperformed earlier tools for post-ASCT outcome prediction [17], Tentori et al. developed a genomic-based algorithm to optimize transplant timing [18], and an EBMT consensus report confirmed that IPSS-M-defined clinical-genomic profiling should guide the indication and timing of transplantation rather than blast percentage alone [19].

Despite this progress, the dynamic relevance of IPSS-M before transplantation, specifically whether cytoreductive therapy can improve molecular risk and outcomes, remains uncertain. Building on the observations by Scheid et al. [13], we analyzed a consecutive cohort of MDS patients to determine whether IPSS-M downstaging prior to ASCT influences ASCT-related outcomes.

## Methods

### Study design and patient cohort

We performed a retrospective, single-center study including 128 consecutive adult patients diagnosed with MDS who underwent ASCT at the University Hospital of Cologne between 2013 and 2024 and had at least three months of follow-up. The distribution of transplantation years was comparable between groups, with approximately two thirds of patients in both cohorts transplanted before 2020 and one third thereafter, indicating substantial temporal overlap. Only patients with available cytogenetic and molecular genetic data were included. Diagnostic classification at the time of transplantation followed the WHO 2022 criteria. IPSS-M categories were determined both at initial diagnosis and immediately prior to transplantation.

Patients were stratified into two groups according to treatment sequence: those who underwent frontline ASCT without prior disease-modifying therapy, and those who received pretreatment with hypomethylating agents with (*n* = 7) or without venetoclax (*n* = 24), or intensive chemotherapy (*n* = 10). Intensive chemotherapy consisted of cytarabine/anthracycline-based regimens. The decision to give pretreatment or proceed directly to ASCT was not based on a predefined institutional algorithm but was depended on the discretion of the treating physician and the referring center. Local practice patterns and individual physician preference therefore played a major role, and baseline clinical and disease characteristics of both groups were broadly similar, including age, comorbidity, blast percentage, cytogenetic abnormalities, and IPSS-M risk distribution (Table 1).

**Table 1:** Baseline clinical, cytogenetic, and transplant-related characteristics of the study cohort.

		Overall	Therapy + ASCT	Frontline ASCT	*P*-value
Number of patients		128	41	87	
Age at transplantation, years, median (IQR)		62 (57–68)	64 (60–70)	61 (55.5–66.5)	0.11
Sex, *n* (%)					0.68
	Female	47 (36.7)	14 (34.1)	33 (37.9)	
	Male	81 (63.3)	27 (65.9)	54 (62.1)	
Bone marrow blasts at diagnosis, %, median (IQR)		10.5 (5.0–15.0)	10.0 (5.0–14.0)	11.0 (5.8–15.0)	0.60
11–20% BM blasts at diagnosis, *n* (%)		73 (56.6)	23 (56.1)	50 (56.8)	1.00
Aberrant karyotype, *n* (%)		55 (42.6)	19 (46.3)	36 (41.4)	0.60
HCT-CI at transplantation, median (IQR)		3 (1–4)	3 (1–4)	2 (1–4)	0.67
Conditioning regimen, *n* (%)	Bu/Cy	2 (1.6)	0 (0.0)	2 (2.3)	
	FLAMSA/Clax	2 (1.6)	1 (2.4)	1 (1.1)	
	FLAMSA/Mel	1 (0.8)	1 (2.4)	0 (0.0)	
	FLAMSA/TBI	5 (3.9)	1 (2.4)	4 (4.6)	
	FLAMSA/Treo	26 (20.3)	8 (19.5)	18 (20.7)	
	Flu/Bu	39 (30.5)	15 (36.6)	24 (27.6)	
	Flu/Bu/Mel	1 (0.8)	0 (0.0)	1 (1.1)	
	Flu/Cy	1 (0.8)	1 (2.4)	0 (0.0)	
	Flu/Treo	44 (34.4)	12 (29.3)	32 (36.8)	
	Flu/Treo/Mel	7 (5.5)	2 (4.9)	5 (5.7)	
Inpatient days after ASCT, median (IQR)		25.0 (21.0–30.0)	25.0 (21.0–29.0)	25.5 (20.3–30.5)	0.97
Stem cell source, *n* (%)	BM	2 (1.6)	0 (0.0)	2 (2.3)	
	PBSC	124 (96.9)	41 (100.0)	83 (95.4)	
	MRD	27 (21.1)	10 (24.4)	17 (19.5)	
	MUD	68 (53.1)	22 (53.7)	46 (52.9)	
	MMUD (9/10)	28 (21.9)	8 (19.5)	20 (23.0)	
	Haplo	5 (3.9)	1 (2.4)	4 (4.6)	
HLA matching, *n* (%)	10/10	95 (74.2)	32 (78.0)	63 (72.4)	
	9/10	28 (21.9)	8 (19.5)	20 (23.0)	
	5/10	5 (3.9)	1 (2.4)	4 (4.6)	

*ASCT* allogeneic stem cell transplantation, *BM* bone marrow, *HCT-CI* Hematopoietic Cell Transplantation–Comorbidity Index, *PBSC* peripheral blood stem cells, *Bu* busulfan, *Cy* cyclophosphamide, *Flu* fludarabine, *Treo* treosulfan, *Mel* melphalan, *TBI* total body irradiation, *FLAMSA* fludarabine, amsacrine, cytarabine, *Clax* venetoclax, *MRD* matched related donor, *MUD* matched unrelated donor, *MMUD* mismatched unrelated donor, *Haplo* haploidentical donor.

The primary objective of the study was to determine whether changes in IPSS-M between diagnosis and transplantation were associated with post-transplant outcomes. Secondary objectives were to compare overall survival, progression-free survival, relapse, non-relapse mortality, and graft-versus-host disease outcomes between patients who underwent frontline ASCT and those who received pretreatment before transplantation.

All patients provided written informed consent. The study was approved by the institutional review board and conducted in accordance with the Declaration of Helsinki.

### Diagnostic definitions and disease classification

Diagnoses were established according to the WHO 2017 criteria applicable at the time of transplantation and retrospectively reclassified according to the WHO 2022 and International Consensus Classification (ICC 2022) systems. Cases with 10–19% bone marrow blasts were categorized as MDS with increased blasts-2 (MDS-IB2). According to the current ICC criteria, 8 of 128 patients (6.2%) in our cohort would now be classified as having de novo AML at initial diagnosis. At the time of transplantation, 14 patients had 20–30% bone marrow blasts.

### Molecular profiling and IPSS-M assessment

Genetic and cytogenetic data were obtained from bone marrow samples at diagnosis and immediately prior to transplantation. Targeted next-generation sequencing (NGS) was performed using the Archer™ VARIANPlex™ Core Myeloid assay covering recurrently mutated genes incorporated in the IPSS-M model. Full NGS panel data were unavailable in 12.5% of patients in the upfront ASCT group and 17.7% of patients in the pretreated group. In these cases, IPSS-M assessment was based on available cytogenetic information and targeted single-gene mutation analyses for prognostically relevant alterations, including NPM1, KMT2A, FLT3, and TP53, in accordance with the IPSS-M framework. Importantly, the same molecular data sources were used consistently at diagnosis and prior to transplantation within individual patients, allowing reliable assessment of IPSS-M dynamics over time.

The molecular International Prognostic Scoring System (IPSS-M) was calculated for each patient at two time points: (1) at diagnosis and (2) immediately prior to ASCT. Risk categories (very low, low, moderate low, moderate high, high, very high) were assigned as described by Bernard et al. [16]. IPSS-M downstaging was defined as categorical risk improvement between diagnosis and transplantation.

### Transplantation procedures

Conditioning intensity (myeloablative, reduced intensity, sequential), donor type, stem cell source, and GVHD prophylaxis are summarized in Table 1. GVHD prevention consisted of a calcineurin inhibitor with mycophenolate mofetil in combination with either antithymocyte globulin or post-transplant cyclophosphamide, according to donor type and institutional protocols. Supportive care followed EBMT and local guidelines. Post-transplant maintenance therapy was not routinely administered. No standardized maintenance strategies were applied after ASCT, and post-transplant treatment was limited to relapse management or clinical trial participation.

### Endpoints

OS was measured from the date of allogeneic transplantation to death from any cause or last follow-up. RFS was defined as time to relapse, progression, or death. Graft-versus-host disease/relapse-free survival (GRFS) was calculated according to established consensus criteria, defined as survival without grade III–IV acute GVHD, systemic therapy-requiring chronic GVHD, relapse, or death. Acute and chronic GVHD were graded by standard criteria. Subgroup analyses included patients with MDS-IB2 and those with high or very high IPSS-M risk at the time of transplantation.

### Statistical analysis

Descriptive statistics were used to summarize baseline characteristics. Continuous variables are presented as median with interquartile range (IQR) and were compared using the Mann–Whitney U test, while categorical variables are presented as counts and percentages and were compared using the χ² or Fisher’s exact test, as appropriate. Overall survival (OS) and relapse-free survival (RFS) were estimated by the Kaplan–Meier method and compared using the log-rank test. Median follow-up was estimated using the reverse Kaplan–Meier method. Sensitivity analyses were performed to assess the robustness of survival outcomes, including landmark analyses at the time of transplantation and exclusion of patients with AML-defining genetic alterations. Non-relapse mortality (NRM) and relapse mortality were analyzed as competing events using the Fine–Gray subdistribution hazard model. Cox proportional hazards regression models were used for exploratory analyses of IPSS-M as a continuous variable, including baseline IPSS-M, change in IPSS-M, and treatment strategy as covariates. All statistical analyses were performed using R version 4.4.2 and Python version 3.12.3. A two-sided *p* value < 0.05 was considered statistically significant.

## Results

### Patient characteristics

A total of 128 patients with MDS underwent ASCT, comprising 87 transplanted frontline and 41 who received prior treatment. Baseline characteristics are summarized in Table 1. Median age at transplantation was 60.4 years and was slightly higher in the pretreated group (63.3 vs 59.0 years). The two cohorts were comparable with respect to sex distribution, comorbidity index (comorbidity index (HCT-CI)), and donor type. The median time from diagnosis to transplantation was 14 months in pretreated patients vs. 5 months in the frontline transplant group. Of note, both groups were comparable with regards to the proportion of patients with increased blast counts MDS-IB2 (56.1% vs. 56.8% in pretreatment vs. frontline groups). Aberrant karyotypes were present in 42.6%.

### Genetic landscape at diagnosis

The most frequently mutated genes at initial diagnosis were SF3B1 and TET2, each detected in approximately 16% of patients in both groups (Supplementary Table 1). Other recurrent alterations included ASXL1, SRSF2, RUNX1, DNMT3A, and TP53 (only multi-hit), each occurring in roughly 5–12% of cases. The distribution of mutations was broadly similar between the frontline and pretreated groups.

### Molecular risk distribution and IPSS-M dynamics

Baseline and pretransplant molecular risk categories according to the IPSS-M are summarized in Table 2. At initial diagnosis, most patients were classified as high (29%) or very high (27%) risk. Risk distributions were comparable between the two groups. At the time of transplantation, 61% of patients remained in the high or very high categories, and 8 patients (6%) fulfilled criteria for AML due to blasts above 20%. A categorical improvement in IPSS-M score between diagnosis and transplant was observed in 29%, whereas 41% remained stable and 31% worsened. The frequency of IPSS-M improvement was 34% in the pretreated vs 26% in the frontline ASCT group).

**Table 2:** IPSS-M distribution and changes between diagnosis and transplantation.

		Overall	Therapy + ASCT	Frontline ASCT
*n*		128	41	87
IPSS-M at initial diagnosis, *n* (%)	Very high	35 (27.3)	12 (29.3)	23 (26.4)
	High	37 (28.9)	14 (34.1)	23 (26.4)
	Moderate high	24 (18.8)	4 (9.8)	20 (23.0)
	Moderate low	18 (14.1)	2 (4.9)	16 (18.4)
	Low	13 (10.2)	8 (19.5)	5 (5.7)
	Very low	1 (0.8)	1 (2.4)	0 (0.0)
IPSS-M at transplant, *n* (%)	AML/very high	8 (6.2)	3 (7.3)	5 (5.7)
	Very high	32 (25.0)	8 (19.5)	24 (27.6)
	High	39 (30.5)	16 (39.0)	23 (26.4)
	Moderate high	18 (14.1)	4 (9.8)	14 (16.1)
	Moderate low	18 (14.1)	4 (9.8)	14 (16.1)
	Low	13 (10.2)	6 (14.6)	7 (8.0)
IPSS-M change, *n* (%)	Improved	37 (28.9)	14 (34.1)	23 (26.4)
	Same	52 (40.6)	14 (34.1)	38 (43.7)
	Worse	39 (30.5)	13 (31.7)	26 (29.9)

*ASCT* allogeneic stem cell transplantation, *IPSS-M* Molecular International Prognostic Scoring System, *AML* acute myeloid leukemia.

**Fig. 1:**
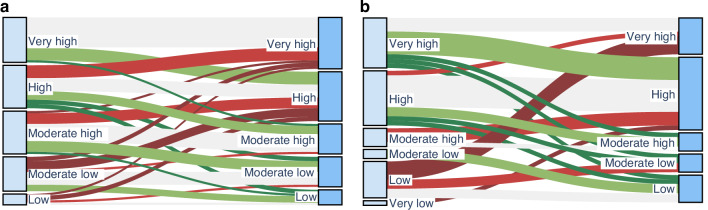
Changes in IPSS-M risk categories from diagnosis to transplantation. Sankey diagrams showing the transitions in IPSS-M risk categories between diagnosis (left, light blue) and transplantation (right, dark blue). The left Panel illustrates the pretreated group, and the right panel shows the frontline ASCT group. Green flows indicate improvement in IPSS-M risk, while red flows represent worsening.

In the pretreated ASCT group, IPSS-M dynamics were evenly distributed, with approximately one third of patients experiencing improved, unchanged or worsened IPSS-M whereas in the frontline ASCT group unchanged IPSS-M was predominant. Changes in IPSS-M categories between diagnosis and transplantation are visualized in Fig. 1 and occurred in both the upfront and pretreatment groups. In patients undergoing upfront ASCT, IPSS-M re-categorization was primarily driven by changes in clinical parameters included in the score, most commonly variations in bone marrow blast percentage and cytopenia severity at reassessment. New cytogenetic abnormalities or newly detected somatic mutations were uncommon in this group and accounted for only a minority of IPSS-M changes.

In contrast, in the pretreatment cohort, IPSS-M reclassification was more frequently associated with molecular or cytogenetic evolution. Interval NGS or cytogenetic reassessment identified newly acquired adverse mutations or expansion of previously low-level clones in a relevant subset of patients, contributing to upward IPSS-M shifts. In several cases, both molecular changes and alterations in blast counts jointly influenced IPSS-M reassignment.

To further quantify the prognostic impact of IPSS-M dynamics, changes in IPSS-M score between diagnosis and transplantation were additionally analyzed as a continuous variable using landmark Cox regression models including baseline IPSS-M and treatment strategy (upfront ASCT vs. pretreatment) as covariates. Increasing IPSS-M from diagnosis to transplantation was significantly associated with inferior RFS and OS, with an ~30% increase in hazard per unit increase in IPSS-M score. In contrast, baseline IPSS-M was not independently associated with outcome in these models. These findings indicate that dynamic molecular risk evolution is a stronger determinant of post-transplant outcome than baseline molecular risk alone (Supplementary Fig. 5).

### Transplant outcomes

**Fig. 2:**
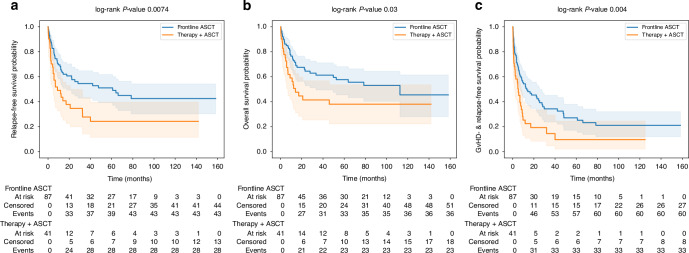
Survival outcomes after frontline versus pretreated ASCT. Kaplan–Meier curves of relapse-free survival (**a**), overall survival (**b**) and graft-versus-host-relapse-free survival (**c**) of the two groups. At-risk tables for each curve are displayed below the plots.

After a median follow-up of 17.3 months, survival outcomes differed between patients undergoing frontline ASCT and those receiving pretreatment when analyzed from the time of diagnosis. Median OS was longer in the frontline group (112.6 months) compared with the pretreated group (14.0 months; *p* = 0.03), and RFS was 61.0 versus 8.9 months, respectively (*p* = 0.007) (Fig. 2).

**Fig. 3:**
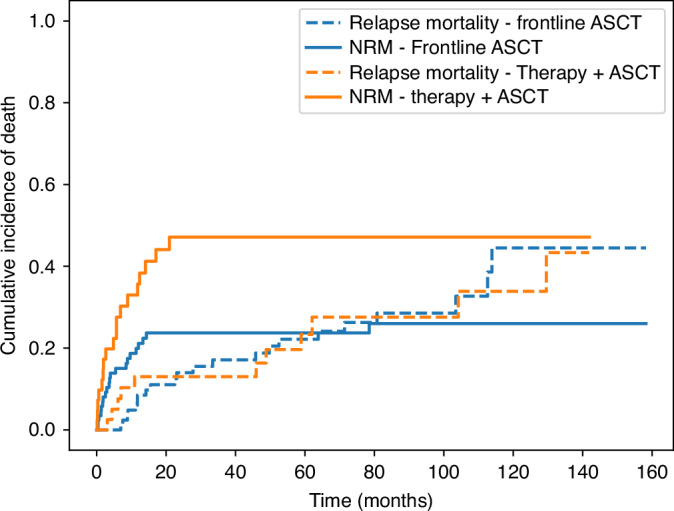
Cumulative incidence curves for relapse mortality and non-relapse mortality in patients undergoing frontline ASCT versus those receiving cytoreductive therapy before ASCT. Relapse mortality and NRM are shown separately for both groups.

A significant difference was also observed in the median GRFS, which was superior in the frontline ASCT group (13.3 vs 5.3 months, *p* = 0.004, Fig. 2). NRM was higher after pretreatment (44.1% vs 23.7%, *p* = 0.018). Relapse mortality was similar in both groups (*p* = 0.99) (Fig. 3). In a landmark analysis starting at the time of ASCT, differences in OS and RFS between upfront and pretreated patients were no longer statistically significant, whereas GRFS remained significantly improved in the upfront group (Supplementary Fig. 2). In a sensitivity analysis excluding patients with AML-defining genetic alterations according to ELN 2022 criteria (NPM1 mutation or KMT2A rearrangement), the results for OS, RFS, and GRFS remained unchanged (Supplementary Fig. 4).

**Fig. 4:**
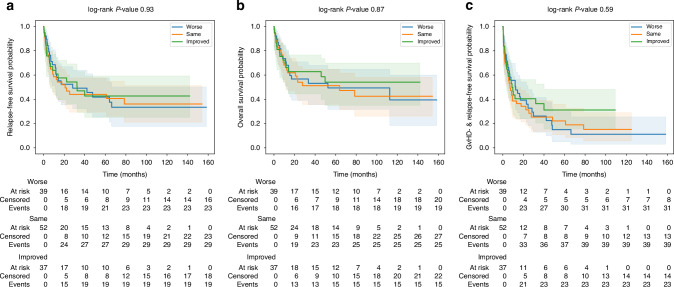
Kaplan–Meier estimates of RFS, OS and GRFS to stratified by IPSS-M change between diagnosis and transplantation. IPSS-M Molecular International Prognostic Scoring System; relapse-free survival (**a**); overall survival (**b**); graft-versus-host disease and relapse-free survival (**c**).

Median duration of hospitalization after ASCT, as well as time to neutrophil and platelet engraftment, were comparable between the two groups. In predefined subgroup analyses, survival outcomes including OS, RFS, and GRFS were similar in patients with increased blasts (MDS-IB2) and in those classified as high or very high IPSS-M risk at diagnosis (Supplementary Fig. 1). To explore whether dynamic changes in molecular risk influenced outcomes, patients were stratified by IPSS-M changes between diagnosis and transplantation into improved, stable, or worsened categories. As shown in Fig. 4, no significant differences were observed in RFS, OS or GRFS between the three groups.

In an exploratory subgroup analysis, patients were stratified according to IPSS-M dynamics between diagnosis and transplantation (improved, stable, worsened). Within each stratum, post-transplant outcomes were compared between patients undergoing frontline ASCT and those receiving cytoreductive therapy prior to transplantation. No significant differences in RFS or OS were observed between treatment strategies across IPSS-M subgroups (Supplementary Fig. 3). The number of patients in each subgroup was limited, and these analyses were not powered to detect modest differences.

## Discussion

The role of cytoreductive therapy before ASCT in MDS remains controversial. While blast reduction has traditionally been pursued to improve remission status and post-transplant outcomes, accumulating evidence suggests that disease biology driven by molecular changes rather than cytoreduction is the principal determinant of prognosis. Unless novel treatments become available, current regimens are unlikely to alter the underlying biology of MDS but may entail toxicity and select for more resistant subclones. In line with this, a large EBMT registry study found no overall benefit of prior therapy in improving IPSS-R before transplantation [13]. Similarly, Schroeder et al. showed that patients undergoing frontline ASCT achieved similar or even better survival than those receiving cytoreduction beforehand, particularly in high-risk subgroups, while GvHD rates were comparable between groups [20].

However, IPSS-R does not account for molecular features and may underestimate disease risk when transplant decisions are made, which led to the development of the IPSS-M. Tentori et al. recently demonstrated that integrating genomic information into transplant timing reclassified ~15% of patients labeled as lower risk by IPSS-R as candidates for immediate transplantation [18]. Complementing these findings, the 2025 international EBMT consensus report by Gurnari et al. confirmed the superiority of IPSS-M and emphasized that transplant timing should be guided primarily by molecular risk rather than morphology alone [19].

Using a cohort of consecutive patients transplanted at a single center with comparable conditioning and GvHD prophylaxis, we evaluated whether cytoreductive pretreatment could meaningfully change the IPSS-M risk profile before ASCT and whether such changes translated into improved post-transplant outcomes. In our cohort, IPSS-M fluctuated modestly in some patients transplanted upfront, but cytoreductive therapy overall failed to reduce molecular risk in the pretreatment group. As shown in Fig. 1, roughly one third of pretreated patients showed improvement, another third remained unchanged, and a substantial subgroup—initially in low or moderate-low IPSS-M categories—shifted to very high risk at the time of transplantation. This pattern supports the concern that cytoreduction may select for fitter, more aggressive subclones rather than stabilizing disease biology. Importantly, the mechanisms underlying IPSS-M dynamics differed between groups, with molecular evolution predominating in pretreated patients and parameter-driven reclassification in upfront transplants.

Another factor that may contribute to the lack of benefit from pretransplant cytoreduction is treatment-related toxicity and delay of transplantation. Although HCT-CI scores at the time of ASCT were comparable between groups, this index may not fully capture dynamic aspects of patient fitness, such as cumulative treatment toxicity, infectious complications, cytopenia burden, or reduced physiologic reserve. In line with this, we observed significantly higher non-relapse mortality and inferior GRFS in the pretreatment cohort (Figs. 2 and 3), suggesting that factors beyond baseline comorbidity influenced transplant outcomes. These observations are consistent with papers demonstrating that additional therapy does not necessarily improve transplant readiness and may compromise the optimal window for ASCT [20–22].

Clonal analyses also support potential harms of pretransplant therapy. In a genomic study of relapsed MDS, Jacoby et al. showed that small preexisting subclones could drive post-transplant relapse and that new structural variants often emerged in expanding clones [21]. Notably, exposure to 5-azacitidine altered the clonal architecture, potentially favoring resistant subpopulations. Consistent with this, a substantial proportion of our pretreated patients transitioned from low or moderate-low IPSS-M at diagnosis to very high risk at ASCT, in some cases due to the acquisition or expansion of adverse mutations, not merely increased blasts. These findings suggest that cytoreductive therapy may not mitigate disease biology and can coincide with adverse molecular evolution.

Beyond biological considerations, omitting pretreatment reduces resource use and may preserve quality of life by avoiding inpatient induction regimens. A similar concept was demonstrated in the randomized ASAP trial for relapsed/refractory AML, a biologically related setting [22]. Immediate transplantation without additional cytoreduction yielded outcomes comparable to intensive reinduction, reinforcing that attempts to optimize morphology before transplant may not provide long-term benefit. These prospective data mirror our findings that short-term cytoreduction does not necessarily translate into durable post-transplant success.

Our study has limitations inherent to its retrospective, single-center design. Subgroup sizes were small, and full NGS panel data were not available for all patients. In cases with incomplete NGS information, molecular risk assessment relied on available cytogenetic data and targeted testing of prognostically relevant genes, which may have reduced granularity of IPSS-M classification. However, this approach was applied consistently at diagnosis and prior to transplantation within individual patients, allowing assessment of IPSS-M dynamics over time. Timing and selection bias are also likely: patients who transformed to overt AML before reaching transplantation were not included, nor were those requiring induction chemotherapy by necessity. Patients transplanted upfront may have benefited from earlier donor identification. Consequently, the intention-to-treat populations for both strategies are not fully reflected by those who ultimately underwent ASCT. These limitations underscore the need for prospective trials that follow patients from diagnosis through donor search and transplantation.

Although we did not identify a subgroup that derived benefit from pretreatment, the question remains whether specific biologically defined subsets might respond favorably to cytoreduction. The impact of different cytoreductive strategies prior to ASCT could not be meaningfully assessed due to limited patient numbers. Pretreatment consisted of hypomethylating agents alone, hypomethylating agents combined with venetoclax, or intensive cytarabine- and anthracycline-based chemotherapy. Given the small size of each subgroup, no formal comparative analysis was performed, and outcomes were not stratified by treatment modality. Importantly, no clear signal of differential benefit emerged across cytoreductive approaches, suggesting that the lack of benefit from pretreatment reflects underlying disease biology rather than the choice of cytoreductive therapy.

Patients whose IPSS-M improved before ASCT did not have better OS, RFS, or GRFS than those with stable or worsening scores, though the improved group was small. This does not exclude the possibility that certain intermediate-risk constellations—such as proliferative disease without TP53 or other high-risk mutations—might derive benefit from targeted pretransplant therapy. Our findings instead highlight a precise avenue for future prospective studies: determining whether a biologically selected subset with intermediate IPSS-M but without adverse molecular features could gain measurable advantage from pretreatment before ASCT.

In summary, pretreatment did not improve OS or RFS compared with frontline ASCT when patients were stratified by IPSS-M. Downstaging of IPSS-M before transplantation, largely driven by blast reduction, did not translate into clinical benefit, reinforcing that baseline molecular risk—rather than short-term cytoreduction—is the major determinant of transplant success. Arbitrary blast thresholds appear less relevant than underlying clonal architecture and its dynamics. These findings argue for relying on biological disease behavior rather than morphologic blast counts when determining transplant timing. In clinical practice, rapid donor identification and proceeding to ASCT once a compatible donor is available may prevent disease evolution and preserve transplant fitness. Prospective studies incorporating IPSS-M–based decision algorithms are warranted to refine molecularly guided strategies for transplant timing in MDS.

## Supplementary information

Supplementary Material

## Data Availability

On reasonable and approved requests made to the corresponding author, data can be shared through secure online platforms.
