# Supplementary material for: IPSS-M downstaging before transplantation does not improve the prognosis of patients with myelodysplastic neoplasms
Source: Bone Marrow Transplant. 2026 Mar 31;61(5):584–90. doi: 10.1038/s41409-026-02845-w (PMC13152802; doi:10.1038/s41409-026-02845-w)

**Supplement**

**Supplementary Table 1:** Ten most frequent gene mutations detected at initial diagnosis in the frontline ASCT and pretreated ASCT groups


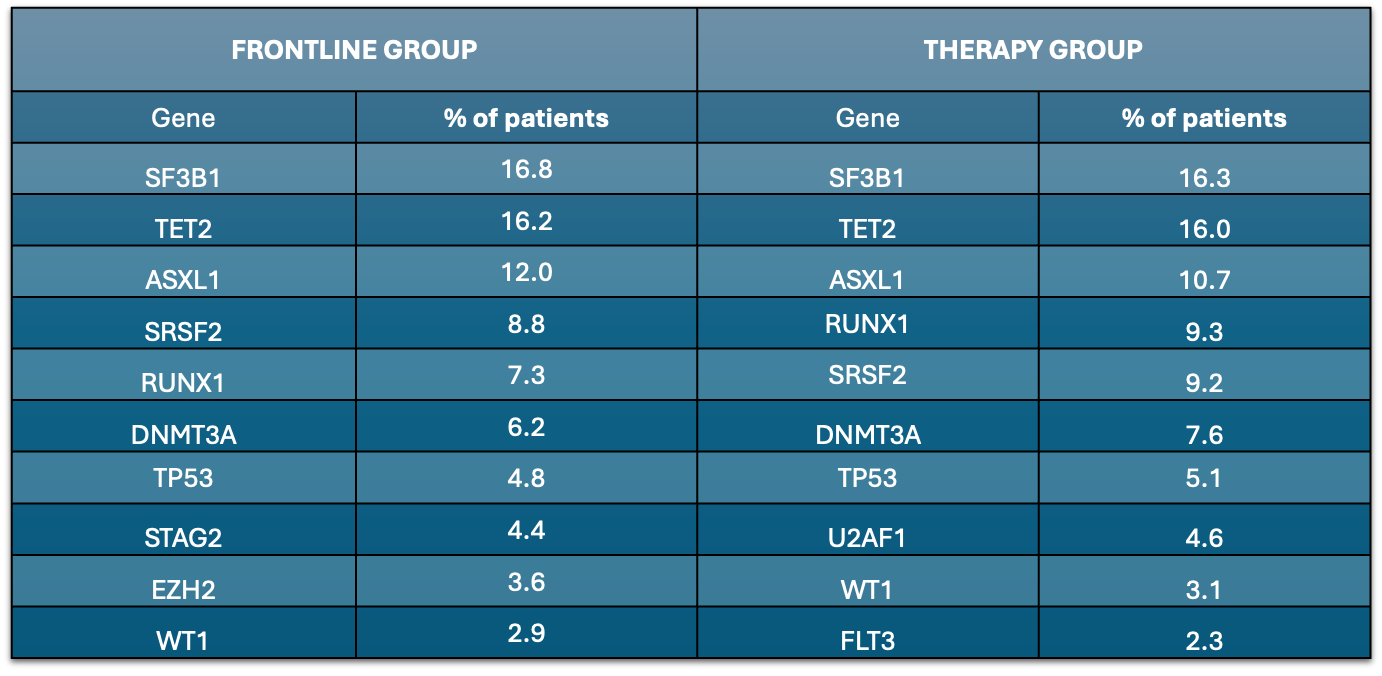


**Supplementary Figure 1:** Kaplan–Meier estimates of relapse-free survival (A+D), overall survival (B+E) and graft-versus-host disease–free, relapse-free survival (C+F) in subgroups with high/very-high IPSS-M (top row) and MDS-IB2 (bottom row) at first diagnosis.


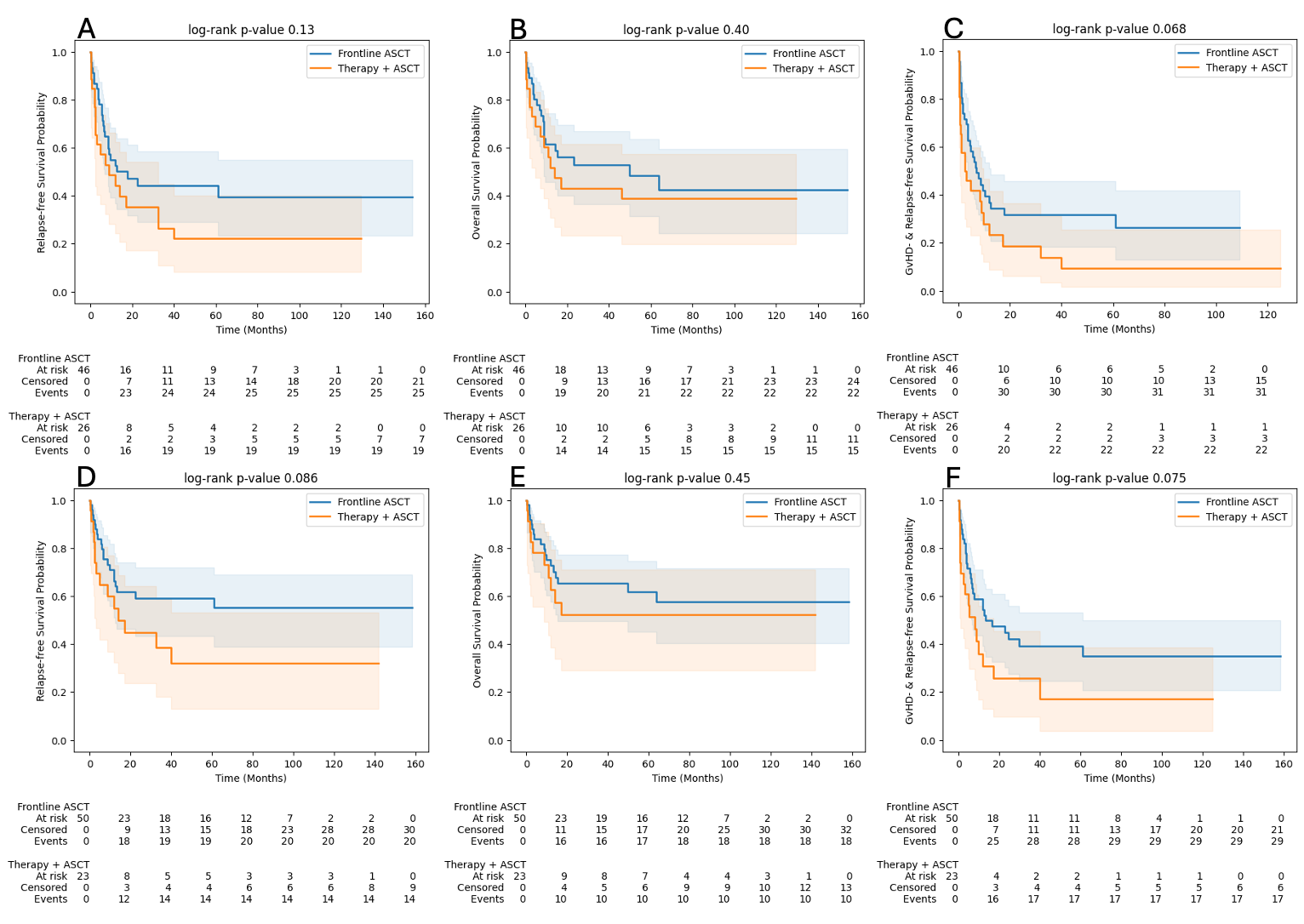


**Supplementary Figure 2:** Landmark Kaplan–Meier estimates of relapse-free survival (A), overall survival (B), and graft-versus-host disease–free, relapse-free survival (C) starting at the time of ASCT in frontline and pretreated patients.


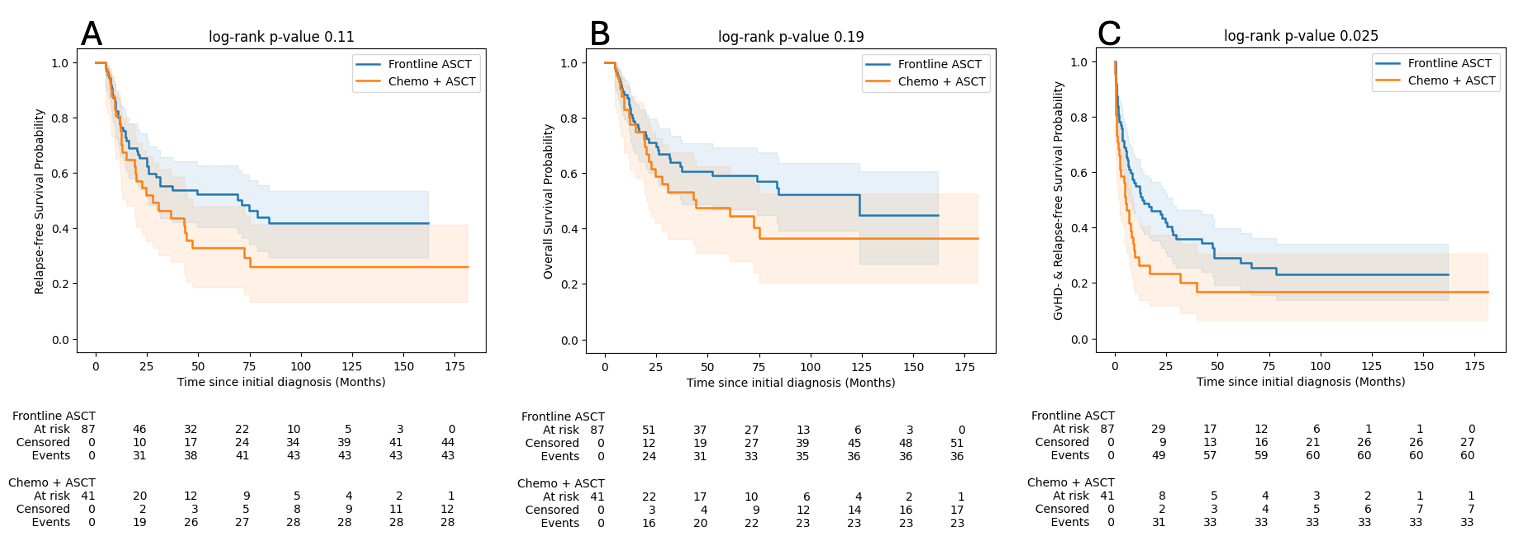


**Supplementary Figure 3:** Kaplan–Meier estimates of relapse-free survival (A) and overall survival (B) stratified by IPSS-M dynamics (worsened, stable, improved) and treatment strategy (frontline ASCT vs pretreatment followed by ASCT). Comparisons between treatment groups within each IPSS-M dynamic category were performed using the log-rank test. Analyses are exploratory and limited by small subgroup sizes.


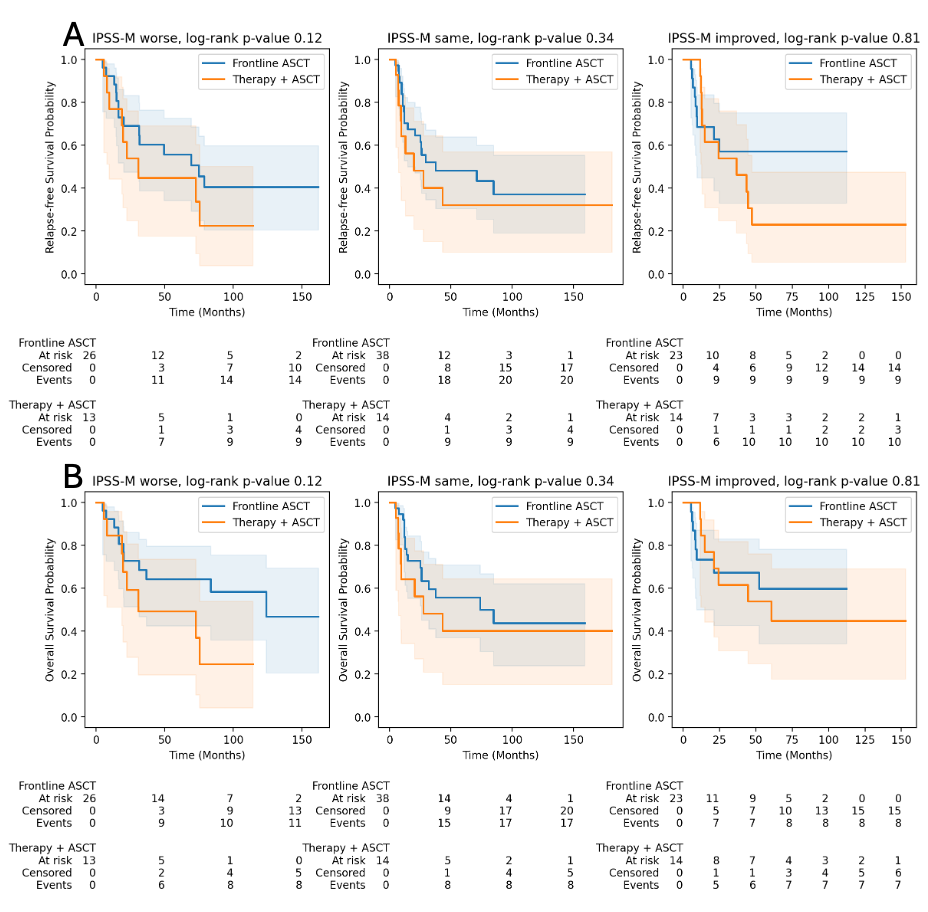


**Supplementary Figure 4:** Kaplan–Meier estimates of relapse-free survival (A), overall survival (B), and graft-versus-host disease–free, relapse-free survival (C) following exclusion of patients with AML-defining genetic alterations (NPM1 mutation or KMT2A rearrangement) according to ELN 2022 criteria. Outcomes are shown for patients undergoing frontline ASCT versus pretreatment followed by ASCT. Shaded areas represent 95% confidence intervals. P values were calculated using the log-rank test.


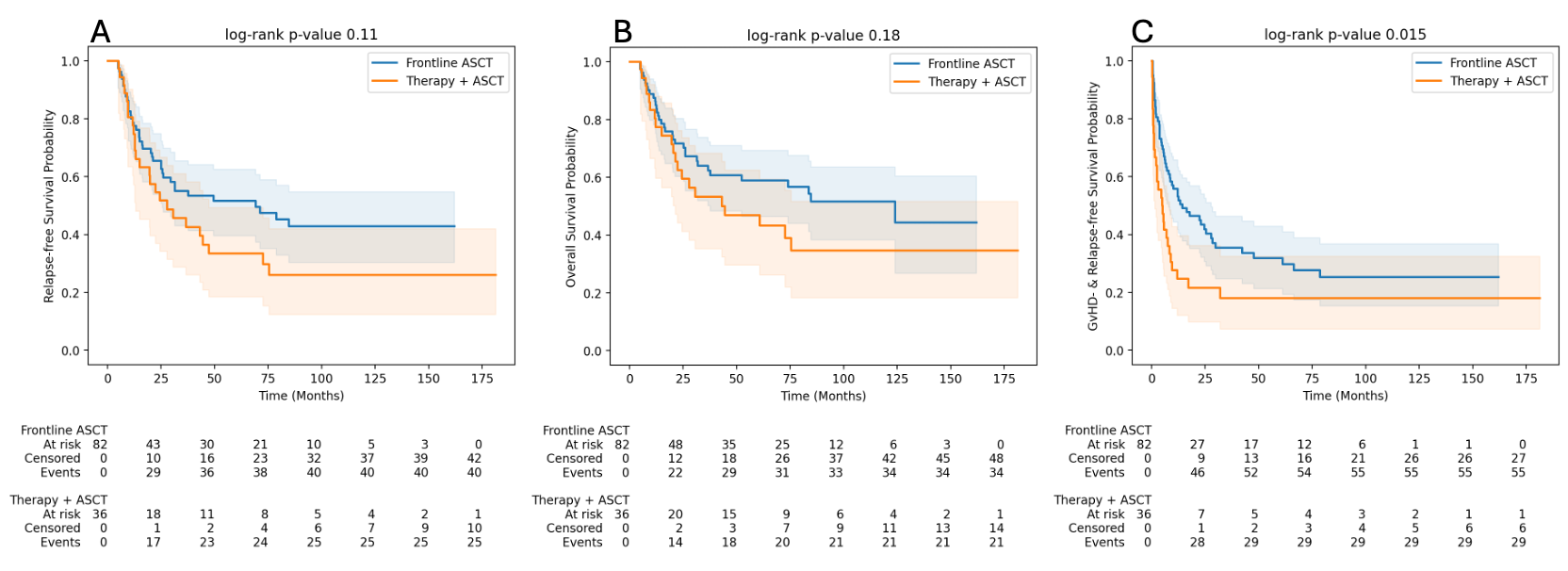


**Supplementary Figure 5:** Landmark Cox regression analyses of relapse-free survival (A) and overall survival (B) including IPSS-M change from diagnosis to transplantation (continuous), baseline IPSS-M score, and treatment strategy (upfront ASCT vs. pretreatment). Increasing IPSS-M change from diagnosis to transplantation was associated with inferior survival independent of baseline risk and treatment strategy, indicating that dynamic molecular progression is a key determinant of outcome.


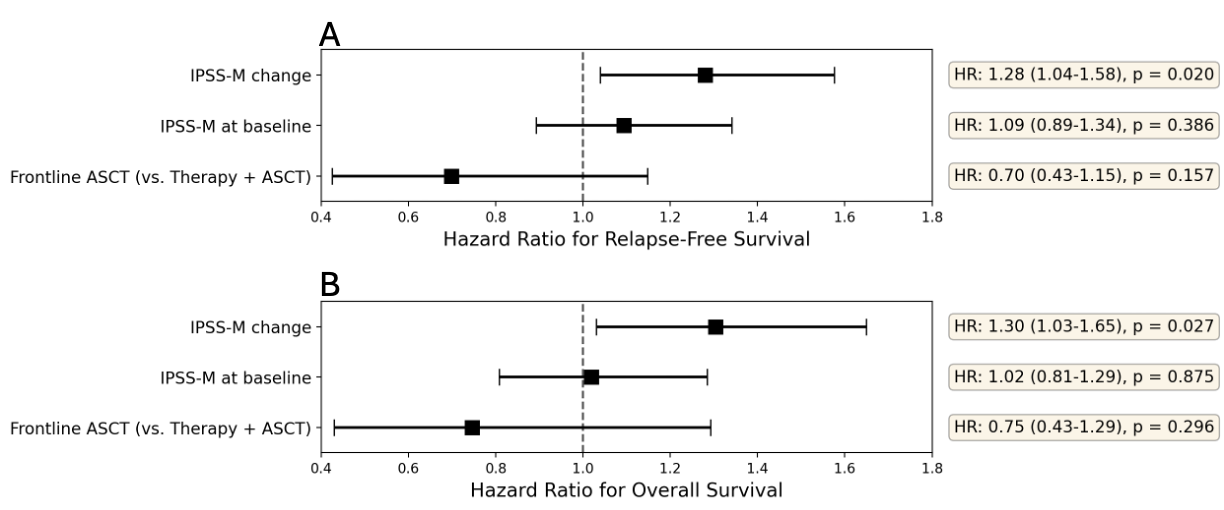

Supplement: Supplementary file 1 — Supplementary Material [file 41409_2026_2845_MOESM1_ESM.docx]
